# New Trends and Advances in MRI and PET Hybrid Imaging in Diagnostics

**DOI:** 10.3390/diagnostics13182936

**Published:** 2023-09-13

**Authors:** Filippo Crimì, Chiara Zanon, Alberto Crimì, Giulio Cabrelle, Emilio Quaia

**Affiliations:** 1 Department of Medicine-DIMED, Institute of Radiology, University of Padova, Via Giustiniani 2, 35128 Padova, Italy; chiara.zanon.5@studenti.unipd.it (C.Z.); giulio.cabrelle@gmail.com (G.C.); emilio.quaia@unipd.it (E.Q.); 2 Orthopedics and Orthopedic Oncology, Department of Surgery, Oncology and Gastroenterology DiSCOG, University of Padova, Via Giustiniani 2, 35128 Padova, Italy; alberto.crimi@aopd.veneto.it

Imaging holds an irreplaceable role in routine clinical practice. Nowadays, almost every clinician for diagnosis needs imaging confirmation and, in recent years, MRI and PET are the two techniques that have experienced the most significant increase in their diagnostic ability, especially in the field of oncology [1,2]. With these concepts in mind, many researchers are developing methodologies and applications to further improve imaging accuracy.

Among these research fields, artificial intelligence, big data, radiomics and texture analysis are being applied in medical images [3]. Using these algorithms, much more hidden information, which cannot be detected by the eye of the radiologist or by a simple qualitative or semi-quantitative evaluation, can be extracted from the row data. In many publications, the results shown are promising and these data suggest that, in the near future, radiologists and clinicians will be aided by specific software both for diagnosis and decision making [4]. This research field is of paramount importance since the workloads in radiology and nuclear medicine departments are ever increasing, and even if different solutions have been proposed to speed up and improve the efficiency of radiologists, it is supposed that the automatic analysis of images is the mainstream solution [5]. Unfortunately, in the radiomics work-flow, the different steps of the analysis involve the potential introduction of biases, each of which would affect the results [4,6]. The principal steps in this workflow are image acquisition, image segmentation, features extraction, data analysis and clinical application, and the significant challenge over the next years will be to standardize these processes as much as possible across different centers in order to establish a reliable technique for imaging analysis [4,6]. Some authors have also shown that artificial intelligence can be useful for generating conclusions from radiological reports, starting from the imaging findings, and, surprisingly, the conclusions reported by the software were generally deemed less harmful and more intelligible by the referring physician than those provided by the radiologist [7,8]. Whilst this might be understood as somewhat endearing, it also serves as a warning that radiologists should drive the change toward these new frontiers so as not to be overwhelmed. This is why research in this field is fundamental.

The standardization of the reporting systems in MRI and PET is another crucial issue, both for accelerating clinical activity in radiology departments and for increasing the reliability of the reports [9]. Many scientific societies are sponsoring this way of reporting and more and more papers are published in this field, sharpening the diagnostic criteria [9]. The necessity of promoting a more subspecialized radiological approach in order to enhance the accuracy of the reports, especially in organ specific examination (e.g., cardiovascular MRI, prostate MRI, etc.), is at the core of this phenomenon.

Other advances in imaging are driven by new radiotracers and new hybrid techniques in nuclear medicine, such as PET/MRI. In nuclear medicine, the continued creation of disease-specific tracers is upgrading the diagnostic accuracy of PET. Beside the most consolidated radiotracers, such as ^11^C- and ^18^F-labeled radiopharmaceuticals, in the last 10 years several novel tracers have been introduced into clinical use for neurological, oncological and inflammatory diseases, showing promising results [10].

A hybrid PET/MRI, due to the fusion of MR and PET images, can combine the highly anatomical details of an MRI with the functional data of the PET images. This is opening new frontiers in different clinical fields, especially in oncology where the combined use of the two techniques can solve myriad problems [11]. In different types of cancers, PET/MRI showed a higher accuracy in identifying the real extent of the disease, the treatment response and the loco-regional and distant metastases compared to the current standard-of-care imaging [11,12,13].

These are only a few of the new trends that are shaping the MR and PET imaging of tomorrow.

The aim of this Special Issue is to highlight the new techniques that can further improve the accuracy and quality of medical imaging by MRI and PET, helping not only clinicians but also radiologists. This Special Issue welcomes full research articles, case reports and reviews focused on advances in MR and PET imaging.
